# Risk factors for early-onset adjacent segment degeneration after one-segment posterior lumbar interbody fusion

**DOI:** 10.1038/s41598-024-59924-5

**Published:** 2024-04-21

**Authors:** Hideaki Nakajima, Shuji Watanabe, Kazuya Honjoh, Arisa Kubota, Akihiko Matsumine

**Affiliations:** Department of Orthopaedics and Rehabilitation Medicine, Faculty of Medical Sciences, University of Fukui, 23-3 Matsuoka Shimoaizuki, Eiheiji-cho, Yoshida-gun, Fukui, 910-1193 Japan

**Keywords:** Outcomes research, Neurological disorders

## Abstract

Adjacent segment degeneration (ASD) is a major postoperative complication associated with posterior lumbar interbody fusion (PLIF). Early-onset ASD may differ pathologically from late-onset ASD. The aim of this study was to identify risk factors for early-onset ASD at the cranial segment occurring within 2 years after surgery. A retrospective study was performed for 170 patients with L4 degenerative spondylolisthesis who underwent one-segment PLIF. Of these patients, 20.6% had early-onset ASD at L3-4. In multivariate logistic regression analysis, preoperative larger % slip, vertebral bone marrow edema at the cranial segment on preoperative MRI (odds ratio 16.8), and surgical disc space distraction (cut-off 4.0 mm) were significant independent risk factors for early-onset ASD. Patients with preoperative imaging findings of bone marrow edema at the cranial segment had a 57.1% rate of early-onset ASD. A vacuum phenomenon and/or concomitant decompression at the cranial segment, the degree of surgical reduction of slippage, and lumbosacral spinal alignment were not risk factors for early-onset ASD. The need for fusion surgery requires careful consideration if vertebral bone marrow edema at the cranial segment adjacent to the fusion segment is detected on preoperative MRI, due to the negative impact of this edema on the incidence of early-onset ASD.

## Introduction

Posterior lumbar interbody fusion (PLIF) is effective for treating lumbar spinal canal stenosis (LSS) with vertebral instability. Use of PLIF has increased with aging of the population and advances in implants and techniques. However, adjacent segment degeneration (ASD) is a well-known postoperative complication associated with PLIF^1^. ASD is classified into radiological, symptomatic, and operative types^2^, with reported incidences of 36–84%, 0–24%, and 0–24%, respectively^3–7^. These incidences vary due to differences in preoperative pathology, fusion levels, and numbers of fused segments, all of which are risk factors for ASD, and an accurate assessment requires consistent definitions of each factor.

ASD after PLIF is generally regarded as a long-term complication, but bimodal peaks for ASD have been reported at 2 and 10 years after primary PLIF^8^. A few studies have focused on the relationship between the influence of risk factors and the time period　before ASD. Several studies have also suggested an association between the incidence of ASD and morphological, surgical, and lumbosacral sagittal alignment factors. Progression of disc degeneration with aging is influenced by the length of the follow-up period^8,9^, whereas early-onset ASD is likely to be affected directly by surgery and/or biomechanical factors without aging-related disc degeneration. Thus, the pathologies may differ in early- and late-onset ASD, but both can be debilitating and unpleasant for patients and may require revision surgery^2,8^.

The relevance of radiological early-onset ASD to clinical outcomes is uncertain^10,11^, but ASD has been suggested to be the most common reason for revision surgery within 1 to 2 years after lumbar interbody fusion surgery^12,13^. Therefore, there is a need for an analysis of risk factors for early-onset ASD in a strictly targeted group of patients with the same preoperative pathology and fusion level, and treatment with the same surgical procedure. The aim of this study was to identify these risk factors in LSS cases treated with one-segment PLIF at L4-5 for L4 degenerative spondylolisthesis.

## Results

### Differences between patients with and without early-onset ASD

A summary of clinical data and imaging findings is shown in Table 1. Of 170 patients who underwent one-segment PLIF at L4-5, early-onset ASD at L3-4 within 2 years after surgery was detected in 35 patients (20.6%). There were no significant differences in background (age, sex, BMI, smoking habit, and preoperative JOA score) between the ASD and non-ASD groups. Imaging assessments showed significantly higher preoperative % slip (*p* = 0.003), although reduction of slip (Δ% slip) did not differ significantly between the two groups (*p* = 0.069). The ASD group had significantly higher rates of a vacuum phenomenon (*p* = 0.002) and bone marrow edema (*p* < 0.001) at L3-4 on preoperative imaging. There were no significant differences in pre- and postoperative spinopelvic alignment. Among factors related to the surgical procedure, cage height (*p* = 0.027), disc space distraction (*p* = 0.003), and concomitant L3-4 decompression (*p* = 0.001) were significantly higher in the ASD group.

**Table 1:** Clinical data and imaging findings in patients with and without early-onset ASD.

Variables	Non-ASD	ASD	*p* Value
Number of patients, n (%)	135 (79.4%)	35 (20.6%)	
Background			
Age, years	68.00 [61.00, 74.50]	68.00 [63.00, 75.50]	0.608
Sex (male/female)	51 (37.8%) / 84 (62.2%)	9 (25.7%) / 26 (74.3%)	0.184
BMI, kg/m^2^	23.30 [21.80, 25.70]	23.30 [21.55, 26.60]	0.489
Smoking habit	36 (26.7%)	7 (20.0%)	0.516
Preoperative JOA score	17.00 [14.00, 19.00]	17.00 [15.00, 19.00]	0.833
Imaging findings			
Preoperative % slip, %	19.00 [14.00, 24.00]	23.00 [19.50, 28.00]	0.003*
Δ% slip, %	14.00 [8.75, 18.55]	17.00 [11.50, 20.30]	0.069
L-DISH	17 (20.7%)	3 (8.6%)	0.769
Preoperative PI, degrees	50.00 [43.00, 56.00]	49.00 [38.75, 52.00]	0.191
Preoperative LL, degree	36.00 [28.00, 43.00]	35.00 [27.00, 40.00]	0.383
Δ LL, degree	0.00 [− 3.50, 3.00]	2.00 [− 4.50, 4.00]	0.737
Preoperative PI-LL, degrees	15.00 [9.00, 21.50]	15.00 [9.00, 22.00]	0.955
Postoperative PI-LL, degrees	15.00 [8.00, 20.00]	15.00 [8.50, 20.00]	0.71
L3-4 vacuum phenomenon	20 (14.8%)	14 (40.0%)	0.002*
L3-4 bone marrow signal change			
bone marrow edema (type I )	18 (13.3%)	24 (68.6%)	< 0.001*
fatty conversion (type II)	19 (14.1%)	1 (2.9%)	0.080
endplate sclerosis (type III)	1 (0.7%)	0 (0.0%)	1
Surgical procedure			
Splitting laminotomy	22 (16.3%)	10 (28.6%)	0.143
PPS	28 (20.7%)	11 (31.4%)	0.184
Cage height, mm	9.00 [8.00, 10.00]	8.00 [8.00, 9.00]	0.027*
Disc space distraction, mm	2.80 [1.60, 3.60]	3.70 [2.75, 5.45]	0.003*
L3-4 decompression	31 (23.0%)	19 (54.3%)	0.001*

Data are shown as median [interquartile] or number (%), *p < 0.05.

BMI, body mass index; JOA, Japanese Orthopaedic Association; L-DISH, diffuse idiopathic skeletal hyperostosis extending to the lumbar segment; PI, pelvic incidence; LL, lumbar lordosis; PPS, percutaneous pedicle screw.

### Differences between patients with radiographic and clinical early-onset ASD

Of the 35 patients with early-onset ASD, 9 (25.7%) developed symptoms within 2 years after surgery and 2 required additional surgery. The median time of symptom onset in these cases was 19 [10.0, 24.0] months after surgery. Preoperative LL was significantly lower in the clinical ASD group than in the radiographic ASD without clinical symptoms group. The other clinical data and imaging findings were not significantly different between two groups (Table 2).

**Table 2:** Clinical data and imaging findings of patients with radiographic and clinical ASD.

	Early-onset ASD		*p* Value
	Radiographic ASD	Clinical ASD (< 2yrs)	
Patient number, n (%)	26 (74.3%)	9 (25.7%)	
Patient background			
Age, years	67.50 [63.25, 75.75]	68.00 [61.00, 75.00]	0.777
Sex (male/female)	20 (76.9%) / 6 (23.1%)	6 (66.7%) / 3 (33.3%)	0.869
BMI, kg/m^2^	23.30 [21.52, 27.00]	23.30 [22.30, 24.60]	0.97
Smoking habit	3 (11.5%)	4 (44.4%)	0.10
Preoperative JOA score	17.00 [16.00, 19.00]	16.00 [11.00, 17.00]	0.07
Image findings			
Preoperative % slip, %	23.00 [19.25, 28.00]	21.00 [20.00, 25.00]	0.895
Δ % slip, %	16.20 [12.00, 19.45]	18.00 [10.10, 22.00]	0.558
L-DISH	1 (3.8%)	2 (22.2%)	0.314
Preoperative PI, degree	49.00 [42.00, 53.50]	48.00 [42.00, 51.00]	0.438
Preoperative LL, degree	36.50 [28.50, 41.50]	25.00 [25.00, 34.00]	0.049*
Δ LL, degree	2.00 [− 4.75, 3.75]	1.00 [− 4.00, 4.00]	0.985
Preoperative PI-LL, degree	13.00 [9.25, 20.00]	19.00 [9.00, 24.00]	0.623
Postoperative PI-LL, degree	15.00 [7.25, 20.00]	19.00 [11.00, 20.00]	0.461
L3-4 vacuum phenomenon	11 (42.3%)	3 (33.3%)	0.937
L3-4 bone marrow signal change			
Bone marrow edema (type I)	18 (69.2%)	6 (66.7%)	1
Fatty conversion (type II)	1 ( 3.8%)	0 ( 0.0%)	1
Surgical procedure			
Splitting laminotomy	8 (30.8%)	2 (22.2%)	0.951
PPS	8 (30.8%)	3 (33.3%)	1
Cage height, mm	8.00 [8.00, 9.00]	8.00 [8.00, 9.00]	0.718
Disc space distraction, mm	3.65 [2.73, 5.33]	4.00 [2.90, 5.50]	0.762
L3-4 decompression	12 (46.2%)	7 (77.8%)	0.21

Data are shown as median [interquartile] or number (%).

BMI, body mass index; JOA, Japanese Orthopaedic Association; L-DISH, diffuse idiopathic skeletal hyperostosis extending to the lumbar segment; PI, pelvic incidence; LL, lumbar lordosis; PPS, percutaneous pedicle screw.

### Prognostic factors for early-onset ASD after one-segment PLIF at L4-5

**Figure 1:**
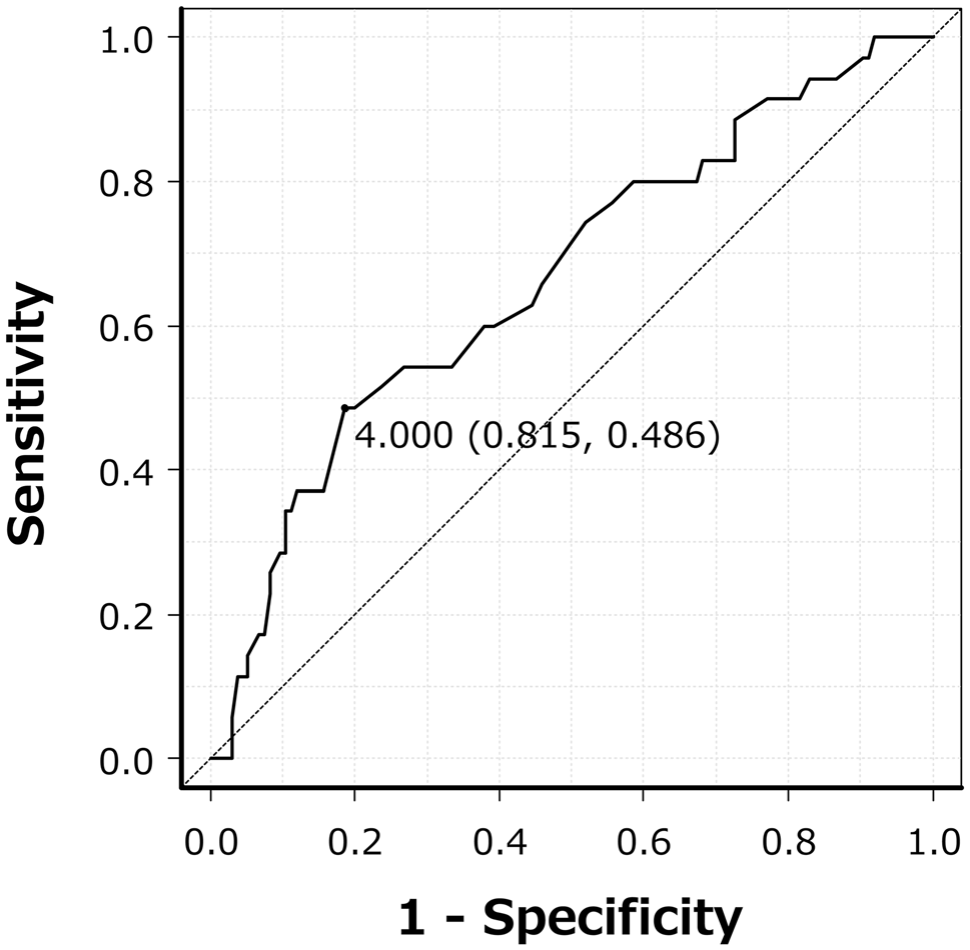
ROC curve of surgical disc space distraction as a predictor of early-onset adjacent segment degeneration (ASD). The curve was used to determine a cut-off value of 4.0 mm.

Multivariate logistic regression analysis including significant variables from univariate analysis, excluding cage height as a confounding factor for disc space distraction, was used to identify prognostic factors in patients with early-onset ASD after one-segment PLIF at L4-5. In this analysis, preoperative % slip (OR 1.08), L3-4 bone marrow edema on preoperative MRI (OR 16.80), and disc space distraction (OR 1.40) were identified as independent prognostic factors (Table 3). The AUC of the ROC curve for disc space distraction was 0.67, and the cut-off for early-onset ASD at L3-4 after one-segment PLIF at L4-5 was 4.0 mm (sensitivity, 48.6%; specificity, 81.5%) (Fig. 1). Vacuum phenomenon and concomitant decompression at L3-4 were not identified as prognostic factors.

**Table 3:** Multivariate logistic regression analysis of risk factors for early-onset ASD.

Variables	Odds ratio	95% CI	*p* Value
Preoperative % slip	1.08	1.01–1.15	0.023*
L3-4 vacuum phenomenon	0.95	0.32–2.84	0.93
L3-4 bone marrow edema	16.80	5.47–51.50	< 0.001*
Disc space distraction	1.40	1.06–1.86	0.018*
L3-4 decompression	2.29	0.87–6.04	0.094

*p < 0.05.

ASD, adjacent segmental degeneration.

### Clinical and imaging features in patients with preoperative vertebral bone marrow edema

Among the 35 patients with early-onset ASD, 24 (57.1%) had findings of vertebral bone marrow edema on preoperative MRI (Table 4). In the non-ASD group, 18 cases (13.3%) had similar findings. Clinical data and imaging findings, age, sex, BMI, L3-4 preoperative disc height, L4-S1 angle, L3-4 vacuum phenomenon, L3-4 vertebral bone marrow edema area, and concomitant decompression at L3-4 were compared for patients with vertebral bone edema with and without ASD. However, no significant predictors of early-onset ASD after one-segment PLIF at L4-5 were found in patients with vertebral bone marrow edema at L3-4 on preoperative MRI.

**Table 4:** Clinical and imaging features in patients with and without early-onset ASD after PLIF at L4-5 among cases with vertebral bone edema at L3-4 on preoperative MRI.

Variables	Non-ASD	ASD	*p* Value
Number of patients, n (%)	18 (13.3%)	24 (57.1%)	
Background			
Age, years	73.50 [64.50, 76.00]	72.50 [63.75, 77.25]	0.899
Sex (male/female)	8 (44.4%) / 10 (55.6%)	7 (29.2%) / 17 (70.8%)	0.347
BMI, kg/m^2^	22.95 [20.55, 26.50]	24.70 [22.08, 27.27]	0.174
Imaging findings			
L3-4 Preoperative disc height	6.10 [4.80, 7.83]	6.35 [5.60, 7.58]	0.703
L4-S1 angle	17.95 [13.17, 22.10]	19.00 [13.70, 24.15]	0.675
L3-4 vacuum phenomenon	9 (50.0%)	13 (54.2%)	1
L3-4 bone marrow edema area	151.35 [108.03, 206.90]	169.76 [115.07, 276.31]	0.477
Surgical procedure			
L3-4 decompression	8 (44.4%)	14 (58.3%)	0.533

Data are shown as median [interquartile] or number (%), *p < 0.05.

ASD, adjacent segmental degeneration; BMI, body mass index.

### Representative cases

**Figure 2:**
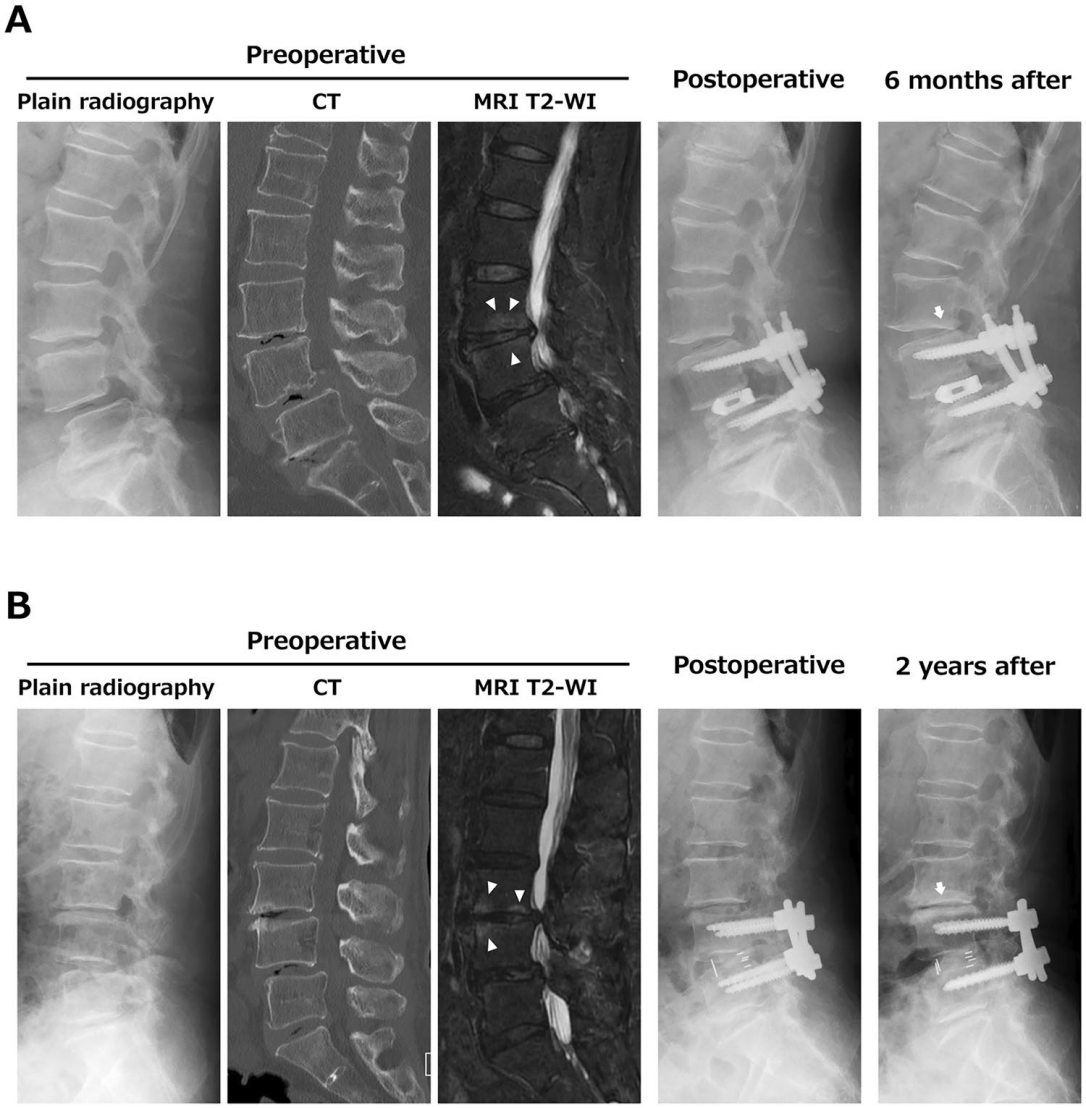
Representative cases. (**A**) A 63-year-old female with a preoperative vacuum phenomenon and vertebral bone marrow edema (arrowheads) at the caudal adjacent segment. At 6 months after surgery, there was a decrease in disc height. (**B**) An 82-year-old female with a preoperative vacuum phenomenon and vertebral bone marrow edema at the caudal adjacent segment (arrowheads). Surgical disc space distraction was 4.0 mm. At 2 years after surgery, the patient had a decrease in disc height with low back pain.

A 63-year-old female underwent PLIF at L4-5 with split laminotomy at L3-4. Preoperative CT and MRI indicated a vacuum phenomenon at L3-4 and L4-5, and bone marrow edema at L3-4. Preoperative % slip was 24%, Δ% slip was 3%, and disc space distraction was 2.4 mm. At 6 months after surgery, a decrease in disc height of > 3 mm was detected (Fig. 2A).

An 82-year-old female underwent PLIF at L4-5 with conventional laminotomy at L3-4. Preoperative CT and MRI showed a vacuum phenomenon at L3-4, L4-5 and L5-S1 and bone marrow edema at L3-4. Preoperative % slip was 18%, Δ% slip was 13%, and disc space distraction was 4 mm. At 2 years after surgery, the patient had a decrease in disc height of > 3 mm with low back pain (Fig. 2B).

## Discussion

The aim of this study was to assess risk factors for early-onset ASD at the cranial segment following one-segment PLIF at L4-5. Of the patients in the study, 20.6% had early-onset ASD within 2 years after surgery. Preoperative larger % slip, vertebral bone marrow edema at the cranial adjacent segment on preoperative MRI, and surgical disc space distraction (cut-off: 4.0 mm) were identified as predictors of early-onset ASD. In contrast, there was no independent association of a preoperative vacuum phenomenon at L3-4, simultaneous partial laminectomy at L3-4 for concomitant canal stenosis, surgical reduction of slip (Δ% slip), or pre-/postoperative spinopelvic alignment with early-onset ASD.

Several risk factors for ASD following PLIF have been described, including older age (≥ 60 years), genetic factors, high BMI (≥ 25 kg/m^2^), and history of smoking^7,14–17^. In the current study, there was no association between these factors and early-onset ASD, although we note that the median age of 68.0 years was high. In imaging, preoperative % slip was an independent risk factor for early-onset ASD. However, this factor cannot be controlled and the more important finding was that Δ% slip was not associated with early-onset ASD. L-DISH has been suggested to be a risk factor for ASD and/or pseudoarthrosis following fusion surgery. ASD-free survival is 93.8% and 90.1% at 5 and 10 years after surgery, respectively^18^, whereas ASD-free survival in L-DISH cases was significantly lower in the early phase after surgery, at 78% at 12 months and 63% at 24 months^19^. An association of early-onset ASD with PLIF at a lower segment and one-segment distant from the segment adjacent to L-DISH has been suggested. Lumbar spinous process-splitting laminotomy may also have a negative effect, especially with decompression at a lower segment adjacent to L-DISH^20^. The effect of L-DISH is noteworthy, but only a few of our cases had the lower end of L-DISH extending to L3 or L4, which made it difficult to detect a correlation with ASD.

The association between ASD and spinopelvic parameters has been well described, and maintenance of a postoperative L4-5 segmental lordotic angle > 20° may be important to prevent ASD, even in one-segment fusion surgery^4^. In the current study, preoperative LL was compared between patients with early-onset clinical ASD and those with radiographic ASD without clinical symptoms. A previous study suggested that the changes in segmental lordosis was a major risk factor for early-onset ASD. Therefore, it is particularly important to adjust segmental lordosis in patients with early-onset ASD, even in cases of single-segment PLIF surgery^21^. Postoperative PI-LL is also a major risk factor for ASD and segmental lordosis at the fused segment has been associated with postoperative lumbar lordosis^22^. Lumbar hypolordosis increases stress on the posterior spinal column and increases the posterior shear force at the proximal adjacent segment^23,24^. Other studies have linked high PI to an increased risk of early-onset radiological ASD^2^. However, in the current study, preoperative PI and pre-/postoperative PI-LL were not associated with early-onset ASD. These results suggest that maintaining the spinopelvic parameters might be important to prevent both early-onset and late-onset ASD.

In the current study, 57.1% of cases with vertebral bone marrow edema on preoperative MRI had early-onset ASD. To our knowledge, this is the first study to focus on preoperative bone marrow edema at the adjacent segment as a risk factor for ASD, although preoperative findings of vertebral bone marrow edema on MRI have been linked to progression of intervertebral disc degeneration in 1 year after decompression surgery without fusion^25^. A similar pathophysiology may be involved in cranial adjacent intervertebral disorders after one-segment PLIF at L4-5, with or without concomitant L3-4 decompression. Previous pathological studies have shown that bone marrow edema (Modic type I) specimens have higher active bone turnover consistent with bone formation and erosion and the number of inflammatory cells, compared to other Modic types^26,27^. These pathological changes could accelerate disc degeneration, including endplate deformation, decreased disc height and altered disc signal intensity, which are unexplained changes in the natural course^28^. However, no significant patient background factors, imaging findings such as bone marrow edema area, and surgical procedure were found that differentiated cases with and without early-onset ASD among those with bone marrow edema at the cranial segment.

In the surgical procedure, excessive disc space distraction was an independent risk factor for early-onset ASD. In previous studies, excessive disc space distraction caused by insertion of excessively high interbody cages has been associated with increased stress in facet joints and discs at adjacent segments, resulting in degeneration of these segments^29,30^. One study reported L4-5 disc space distraction of 3.1 mm in patients without ASD, compared to 4.4 mm in patients with ASD^31^. Similarly, the cut-off for disc space distraction was 4.0 mm in the current study. This suggests that this cut-off could be a guide to preventing ASD after PLIF, although the sensitivity was low. Laminectomy at the adjacent level of fusion has also been suggested to increase the risk of ASD^32–35^. Thus, preservation of as much of the posterior complex as possible is recommended in cases with adjacent segments that need decompression. However, in the current study, simultaneous partial laminectomy at adjacent segments and less invasive surgical procedures such as split laminotomy and PPS were not associated with the incidence of early-onset ASD.

The study has several limitations, including the retrospective, single-center design, the small number of patients with early-onset ASD, and potential confounding factors for which we were unable to adjust in the analysis. Also, factors that did not show correlations with early-onset ASD in this study may still be important for late-onset ASD. Patients with early-onset radiographic ASD did not necessarily exhibit any symptoms and did not require additional surgery. Therefore, it is further long-term studies are needed to determine the clinical significance of this condition. Despite these limitations, we believe that our findings provide important insights for surgical and clinical management for preventing early-onset ASD after one-segment PLIF at L4-5.

In conclusion, we focused on risk factors for early-onset ASD in this study. Among patients who underwent one-segment PLIF at L4-5, 20.6% had early-onset ASD in the cranial segment within 2 years after surgery. Preoperative larger % slip, vertebral bone marrow edema in the cranial segment on preoperative MRI, and surgical disc space distraction (cut-off: 4.0 mm) were significant independent risk factors for early-onset ASD. Patients with preoperative imaging findings of bone marrow edema in the cranial segment had a 57.1% rate of early-onset ASD, whereas a vacuum phenomenon and/or concomitant decompression at the cranial segment, and the degree of surgical reduction of slippage were not risk factors for early ASD. In addition to risk factors for late-onset ASD, including pre- and postoperative spinal alignment, consideration of the risk factors found in this study may be important for improving surgical outcomes.

## Methods

### Study population and surgical procedures

Between 2005 and 2020, a total of 170 patients (median age 68.0 years; 60 males, 110 females) underwent one-segment PLIF at L4-5 for L4 degenerative spondylolisthesis at University of Fukui Hospital and had a minimum follow-up period of 2 years. Clinical signs and symptoms of LSS at the stenosis level were identified on high-resolution MRI and were not relieved by conservative treatment. Physical examinations indicated motor and/or sensory disturbances in all cases, including bladder dysfunction. PLIF was recommended for patients with spondylolisthesis associated with ≥ 3 mm slippage and/or posterior opening > 5° on dynamic lateral plain radiographs. Those with prior spinal surgery, lumbar trauma, rheumatoid arthritis, destructive spondyloarthritis, neoplasm, infection, lumbar disc herniation, lumbar degenerative scoliosis with a lumbar Cobb angle ≥ 10°, and PLIF at other segments were excluded from this study.

A conventional open midline approach and bilateral total facetectomy were used in all procedures. Disc material was removed and the vertebral endplates prepared, after which two cages were inserted into each intervertebral space with extreme care to preserve the endplates. The cages and intervertebral regions were filled with bone graft material obtained from local autologous bone. Partial laminectomy was performed on patients with concomitant canal stenosis at L3-4. Conventional laminotomy was performed from 2005 to 2014, and lumbar spinous process-splitting laminotomy from 2015 to 2020^36^. Pedicle screw fixation was performed with an open or percutaneous approach.

Before surgery, written informed consent was obtained from each patient. The protocol for a single-center study was approved by the Human Ethics Review Committee of Fukui University Medical Faculty (Approval Number 20220210) and adhered to the Clinical Research Guidelines of the Ministry of Health, Labor, and Welfare of the Japanese Government.

### Outcomes and radiological measurements

Information for age, gender, body mass index (BMI), smoking habit, laminectomy adjacent to the fused segment (L3-4), and preoperative neurological assessments based on the Japanese Orthopaedic Association (JOA) score was extracted from medical records. Radiological studies included plain dynamic lumbar radiography, computed tomography (CT) and high-resolution MRI. Spinopelvic alignment was determined using measurements of the pre- and postoperative lumbar lordotic angle (LL) and the pelvic incidence (PI) on plain lumbar radiographs. LL is the angle between tangents to the L1 superior endplates and S1 superior endplates; and PI is the angle between a line joining the center of the upper S1 endplate to the axis of the femoral heads and a line perpendicular to the upper S1 endplate. Pre- and postoperative % slip were measured and the reduction of slip was calculated (Δ% slip = preoperative % slip − postoperative % slip). Radiographic ASD was defined based on > 3 mm antero- or retrolisthesis, > 3 mm decrease in disc height, or the appearance of symptomatic canal stenosis (ASD group)^31^. Early-onset ASD in this study was defined as cases in which radiological ASD occurred at L3-4 within 2 years after initial PLIF at L4-5. In addition, patients who experienced a recurrence of symptoms within 2 years after surgery such as back pain or leg pain within 2 years after surgery—such as back pain or leg pain—were defined separately as clinical ASD. All other cases were included in the non-ASD group.

The presence of an intradiscal vacuum phenomenon at the cranial segment adjacent to the fused segment was evaluated on sagittal and/or axial CT. DISH was diagnosed on plain lateral X-rays and CT of the lumbar spine below T10 based on the Resnick criteria, which include the presence of ossification/calcification along the anterior aspect of at least 4 contiguous vertebra, relative preservation of the intervertebral disc height, and absence of sacroiliac joint erosion^37^. Contiguous ossification/calcification was assessed using the Mata scoring system^38^. Only grade 3 cases (complete bridging of the disc space) were included in the study; grade 1 (without bridging) and grade 2 (incomplete bridging) were excluded. L-DISH cases were defined as those with DISH bridging to the lumbar segment.

The presence of signal intensity changes of vertebral bone marrow was classified based on T1-weighted imaging (WI)/T2-WI MRI as a so-called Modic type I (low/high intensity), type II (high/iso or high intensity), or type III (low/low intensity) change. This classification represents three clinical states: bone marrow edema (type I), fatty conversion (type II), and endplate sclerosis (type III)^39^. For a type I change, a picture archiving and communication system was used to measure the largest area of vertebral bone edema (mm^2^) in the upper and lower vertebra in the T2-WI parasagittal plane^25^. Measurements were made in triplicate by two observers and the average was used in subsequent analyses.

### Statistical analysis

Data are presented as the median [interquartile range]. Categorical variables were evaluated by Mann–Whitney U-test or chi-square test at a significance level of *p* < 0.05. Factors that were significant in univariate analysis were used in a multivariate regression model. The estimated odds ratio (OR) and 95% confidence interval (CI) were calculated to identify independent predictors of radiographic early ASD within 2 years after surgery. Inter- and intraobserver reliabilities for radiological parameters were assessed using intraclass correlation coefficients (ICCs); ICC (1,3) and ICC (2,3) > 0.75 were taken to indicate good to excellent reliability. The cut-off was defined as the point nearest to the upper-left corner of a receiver operating characteristic (ROC) curve, and the area under the curve (AUC) was used to assess the predictive accuracy of the parameter. All analyses were performed with EZR (Saitama Medical Center, Jichi Medical University, Saitama, Japan)^40^, a GUI for R (The R foundation for Statistical Computing, Vienna, Austria).

### Ethics declarations

The study protocol was approved by the Human Ethics Review Committee of Fukui University Medical Faculty (Approval Number 20220210) and strictly followed the Clinical Research Guidelines of the Ministry of Health, Labor, and Welfare of the Japanese Government.

## Data Availability

Data generated and analyzed during this study are included in this published article. Data and materials are available from the corresponding author subject to reasonable request and subject to the ethical approvals in place and materials transfer agreements.
